# γδ T cell characterisation in the long term after haematopoietic stem cell transplantation and its impact on CMV control and cGVHD severity

**DOI:** 10.1002/cti2.70027

**Published:** 2025-03-07

**Authors:** Faisal Alagrafi, Arwen Stikvoort, Ahmed Gaballa, Martin Solders, Olle Ringden, Thomas Poiret, Lucas CM Arruda, Michael Uhlin

**Affiliations:** ^1^ Department of Medicine Huddinge Karolinska Institutet Stockholm Sweden; ^2^ Healthy Aging Research Institute King Abdulaziz City for Science and Technology (KACST) Riyadh Kingdom of Saudi Arabia; ^3^ Department of Biochemistry and Molecular Biology, National Liver Institute Menoufia University Shebin Elkom Egypt; ^4^ Center for Cell Therapy and Allogeneic Stem Cell Transplantation, Karolinska Comprehensive Cancer Center Karolinska University Hospital Stockholm Sweden; ^5^ Division of Pediatrics, Department of Clinical Science, Intervention and Technology (CLINTEC), Translational Cell Therapy Research (TCR) Karolinska Institutet Stockholm Sweden; ^6^ Department of Immunology and Transfusion Medicine Karolinska University Hospital Stockholm Sweden

**Keywords:** allogeneic haematopoietic stem cell transplantation, cytomegalovirus, graft‐versus‐host diseaseγδ T cell reconstitution

## Abstract

**Objectives:**

The clinical outcome after allogeneic haematopoietic stem cell transplantation (aHCT) relies greatly on the efficient recovery of T cells. Several studies have investigated the short‐term γδ T cell reconstitution and their role in clinical outcomes following haematopoietic stem cell transplantation. Nevertheless, their long‐term characteristics and impact have remained largely unknown.

**Methods:**

We analysed γδ T cells from 20 recipient/donor pairs at phenotypic, clonotypic and functional levels to assess their reconstitution ≥ 8 years (median 18 years) post‐transplantation using high‐parameter flow cytometry and next‐generation sequencing of the TCR γ‐chain.

**Results:**

γδ T cells displayed comparable phenotypic characteristics between recipients and matching donors. The Vδ2^+^ subset showed a more activated phenotype and cytokine production, while the Vδ1^+^ and non‐Vδ2 T cells maintained long‐term CMV control. TCR γ‐chain composition in long‐term survivors was largely restored, with no significant differences in gene segment usage or diversity. A small cohort of recipients with severe chronic graft‐versus‐host disease (GVHD) showed overrepresented donor‐derived private clonotypes. Furthermore, we also found elevated HLA‐DR^+^Vδ1^+^ T cells in recipients with severe chronic GVHD.

**Conclusion:**

Overall, γδ T cells reconstitute with a normalised repertoire, high functional capacity and sustained CMV control ability. An increased proportion of activated Vδ1^+^ T cells correlates with chronic GVHD severity, indicating a potential therapeutic target.

## Graphical abstract

In this study, we found that long‐term γδ T cell reconstitution post allogeneic haematopoietic stem transplantation (aHCT) reached a homeostatic state with a normalised TRG CDR3 repertoire. Importantly, γδ T cells remained functional and provided a long‐term control on cytomegalovirus (CMV) after aHCT. Additionally, some recipients exhibited overrepresented clonotypes and increased HLA‐DR expression on Vδ1 T cells, which are associated with severe chronic graft‐versus‐host disease (cGVHD).

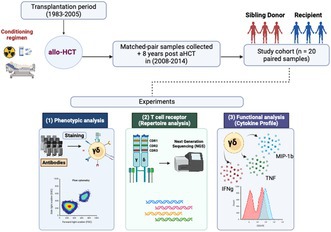

## Introduction

Allogeneic haematopoietic stem cell transplantation (aHCT) represents a potential cure for life‐threatening haematological disorders.^1^, ^2^, ^3^ A critical factor for the success of aHCT is the thorough reconstitution of the ablated immune system, with a rapid T cell recovery being linked to less morbidity and mortality after transplantation.^4^, ^5^ The innate immune system is reconstituted within months, whereas the adaptive system takes years to reach full competence.^6^, ^7^ Shortly after aHCT, the T cell reconstitution is dominated by the peripheral expansion of donor T cells within the graft.^8^ However, these cells offer incomplete protection against opportunistic pathogens because of a limited T cell receptor (TCR) repertoire.^8^, ^9^ Long‐lasting T cell reconstitution is inherently slower, relying on the functionality of the thymus to achieve functional immune homeostasis.^10^ The thymic‐dependent pathway involves the *de novo* generation of T cells, leading to a more diverse T cell repertoire.^10^, ^11^, ^12^, ^13^ Although extensive research has been conducted on the reconstitution of αβ T cells following transplantation,^14^, ^15^, ^16^ there is less information on the recovery of γδ T cells and their clinical implications.

γδ T cells are a distinct and heterogeneous population that represent less than 10% of peripheral T cells in adults, although they are more frequent in tissue sites.^17^ Human γδ T cells are divided into several subsets based on the δ chain, of which Vδ1 and Vδ2 are the prevalent subsets. Different from αβ T cells, γδ T cells possess innate and adaptive immunity.^17^ They express a group of NK‐ and Toll‐like receptors^17^, ^18^ and exert a rapid response against transformed cells.^17^, ^19^ Early after aHCT, γδ T cells start reconstituting rapidly within a few weeks.^20^, ^21^ This rapid reconstitution is involved in several crucial functions, such as antitumor activity and infection control.^22^, ^23^ Additionally, a diverse intestinal microbiome and their metabolites appear to support the early Vδ2 T cell reconstitution.^24^ Patients with a high number of donor‐derived γδ T cells after aHCT have a significantly higher overall and leukaemia‐free survival without increasing the risk of acute GVHD.^22^, ^25^, ^26^ Furthermore, increased levels of γδ T cells, mainly Vδ1^+^, during the first months after aHCT are associated with a protective function against viral infection, particularly cytomegalovirus (CMV).^27^, ^28^, ^29^ This early protection is driven by the rapid expansion of distinct clones in response to CMV reactivation,^20^ but little is known about their impact on the long‐term complications and the long‐term stability and diversity of the γδ T cell repertoire has not been described to date.

This study comprehensively analysed the γδ T cell reconstitution in a long‐term post‐aHCT setting to address the association between γδ T cell composition and functionality with clinical outcome. By analysing 20 recipient/donor pairs at least 8 years after transplantation, we found that γδ T cells in recipients and donors showed similar phenotypic features, with some notable differences in the Vδ2 subset, which presented higher expression of NKG2D, CD39, HLA‐DR, PD‐1 and cytokine production. Additionally, we observed a correlation between CMV serostatus/reactivation and the ratio of Vδ1^+^ to non‐Vδ1/Vδ2 T cells, indicating that these cells may be important for long‐term CMV control. The TCR γ‐chain (TRG) composition was largely restored, with similar gene segment usage and diversity between donors and recipients. However, recipients with severe chronic GVHD exhibited overrepresented donor‐derived private TRG clonotypes and elevated HLA‐DR^+^ Vδ1 T cells. These findings suggest that, while γδ T cells generally reconstitute with a normalised repertoire and sustained CMV control, an increased proportion of HLA‐DR^+^ Vδ1 T cells might be linked to chronic GVHD, indicating a possible therapeutic target.

## Results

### Clinical characteristics of long‐term aHCT patients

The recipients and donors median age at the time of aHCT was respectively 41.5 and 43 years. The median sampling interval/duration of recipients/donors was 18/18.5 years post‐aHCT (Supplementary figure 1). Additional clinical information and outcomes of the recipients are shown in Table 1.

**Table 1:** Clinical characteristics

	Recipients	Donors
Total number	20	20
Gender: female/male, *n*	4/16	13/7
Median age at time of HSCT (range)	41.5 (1.5–64)	43 (3–60)
< 42 (%)	10 (50%)	8 (40%)
≥ 42 (%)	10 (50%)	12 (60%)
CMV serostatus pre‐HSCT, −/+, *n*	6/14	6/14
D+/R+	11	
D−/R+	3	
D+/R−	2	
D−/R−	4	
Underlying disease, *n*		
AML	7	
CML	6	
MM	2	
MDS/MPS	2	
Myelofibrosis	1	
Lymphoma	1	
Non‐malignant (FA)	1	
Transplantation period range	1983–2005	
Median years of sample collection post‐HCT, (range)	18 (8–29)	18.5 (8–29)
Prior autologous transplantation, *n* (%)	3 (15%)	
Conditioning intensity, *n* (%)		
Myeloablative	15 (75%)	
Reduced intensity	5 (25%)	
GVHD prophylaxis, *n*		
CSA+ MTX	16	
CSA+ MMF	2	
CSA	1	
TCD	1	
ATG: yes/no, *n*	2/18	
Graft source, *n (*%)		
Bone marrow		13 (65%)
PBSC		7 (35%)
NC dose (×10^8^/kg), median (range)	2.2 (0.3–21.5)	
Relapse, *n* (%)	3 (15%)	
CMV reactivation, *n* (%)	14 (70%)	
Time of CMV reactivation post‐HCT, median days (range)	55 (4–6933)	
VZV reactivation *n* (%)	6 (30%)	
aGVHD: 0–I/II–III, *n* Time of aGVHD post‐HCT, median days (range)	12/828 (13–73)	
cGVHD: no‐mild/moderate–severe, *n*	7/13	
cGVHD at time of sampling: moderate–severe, *n*	4/16	
Time of cGVHD post‐HCT, median days (range)	131.5 (80–4067)	

aGVHD, acute graft‐versus‐host disease; AML, acute myeloid leukaemia; ATG, anti‐thymocyte globulin; cGVHD, chronic graft‐versus‐host disease; CML, chronic myeloid leukaemia; CMV, cytomegalovirus; CSA, cyclosporin A; FA, Fanconi anaemia; HSV, herpes simplex virus; MDS, myelodysplastic syndromes; MM, multiple myeloma; MMF, mycophenolate mofetil; MPS, myeloproliferative syndrome; MTX, methotrexate; NC, nucleated cell; PBSC, peripheral blood stem cells; TCD, T cell depletion; VZV, varicella zoster virus.

### γδ T cell reconstitution reached homeostasis and featured high Vδ2 activation

**Figure 1:**
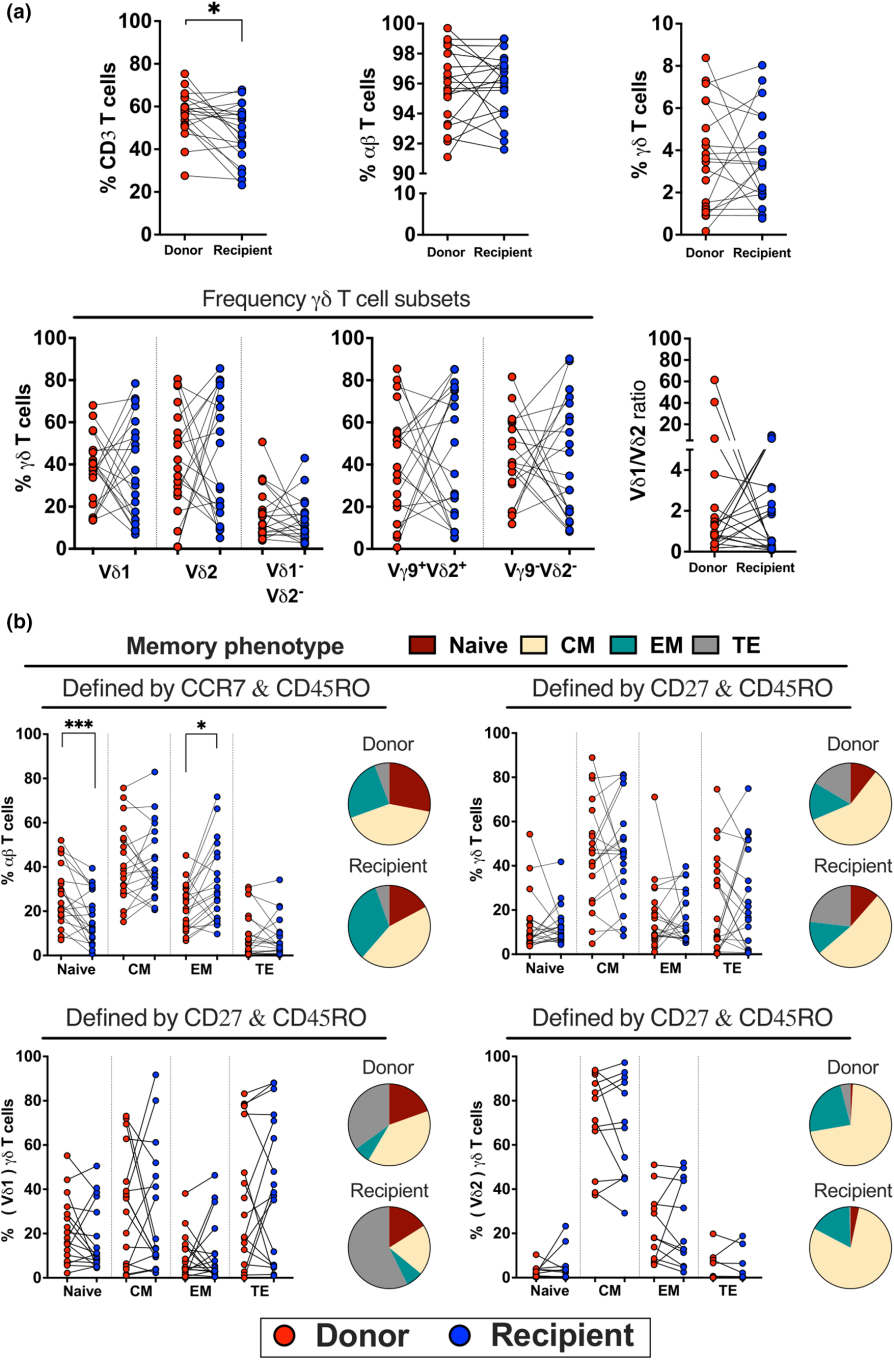
Characterisation of immune cell reconstitution in isolated PBMC of long‐term survivors and their corresponding donors. **(a)** Frequency of CD3^+^ T cells, αβ T cells (gated from γδ neg), and γδ T cells and their subsets, as well as the ratio of Vδ1/ Vδ2 (*n* = 20). **(b)** Memory differentiation markers of γδ T cells and their subsets defined as naïve (CD27^+^CD45RO^−^), central memory (CM: CD27^+^CD45RO^+^), effector memory (EM: CD27^−^CD45RO^+^), and terminally differentiated (TE: CD27^−^CD45RO^−^), whereas the memory phenotype of αβ T cells is defined as naïve (CCR7^+^CD45RO^−^), CM (CCR7^+^CD45RO^+^), EM (CCR7^−^CD45RO^+^) and TE (CCR7^−^CD45RO^−^). Median proportions of memory differentiation stages are depicted in a pie chart. Significant differences were performed by the nonparametric Wilcoxon test, set to *P < 0.05 and ***P < 0.001.

We investigated the phenotypic profile of γδ T cells in long‐term survivors after aHCT (≥ 8 years) and their corresponding donors using flow cytometry (Supplementary table 2). Recipients had a lower frequency of total T cells (median 48.94%, IQR_25‐75_ 38.65–57.38%) compared to donors (median 58.21%, IQR_25‐75_ 51.26–63.02%), but no differences in αβ T cells, γδ T cells, or γδ T cell subsets were observed (Figure 1a). There were no differences in the expression of CD4^+^, CD8^+^, NK cell receptors, chemokine receptors, exhaustion, or activation markers (Supplementary figure 3a–d). In terms of memory phenotype, recipients had a lower proportion of naïve αβ T cells (median 14.55%, IQR_25‐75_ 8.01–30.28%) and a higher proportion of effector memory αβ T cells (median 28.10%, IQR_25‐75_ 16.85–45.90%) than donors (median 25.70%, IQR_25‐75_ 17.88–39.75%; median 22.65%, IQR_25‐75_ 12.80–30.13%, respectively). However, no significant differences were observed in the memory phenotype of γδ T cells or their subsets (Figure 1b).

**Figure 2:**
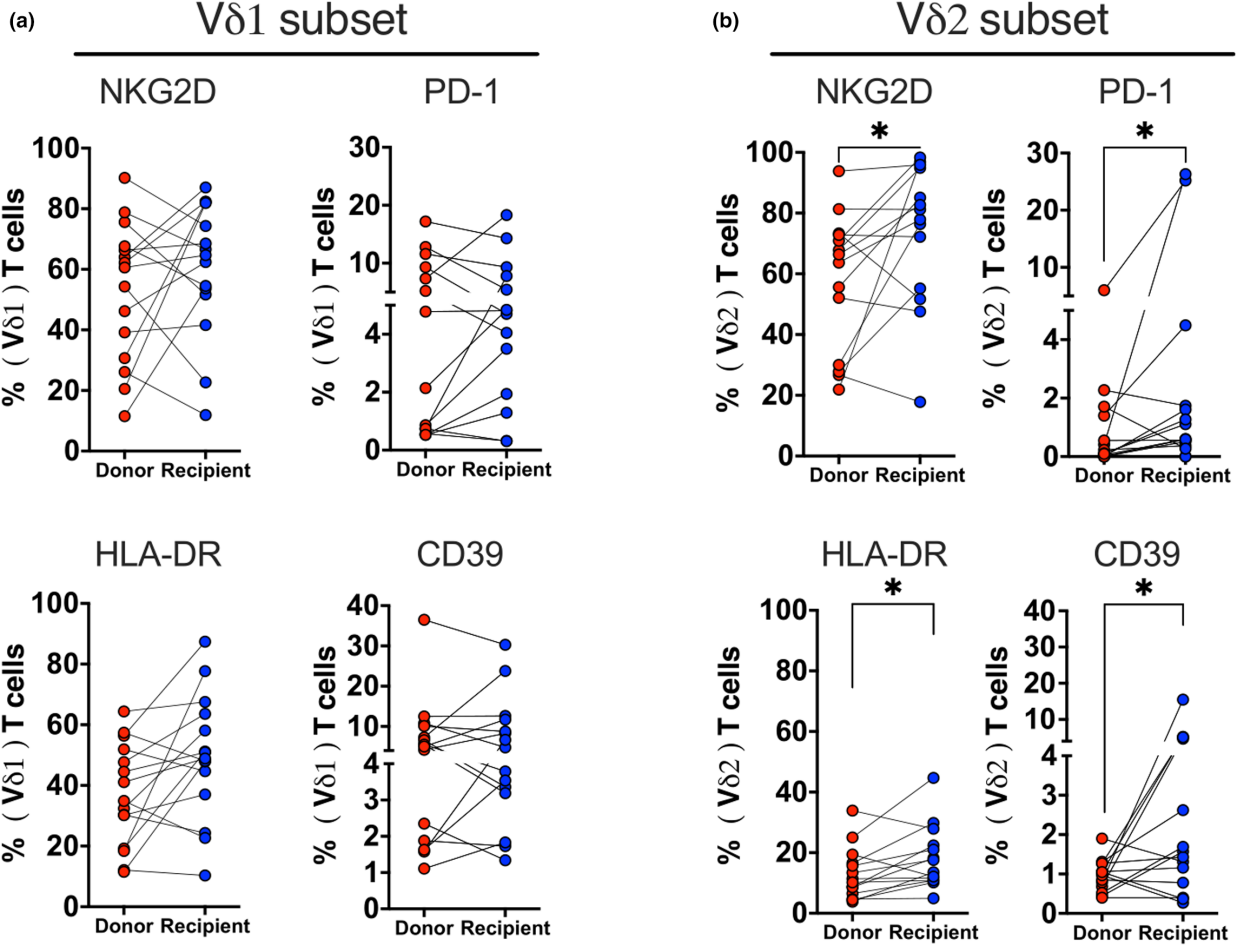
Phenotypic analysis of flow cytometry data for in‐depth characterisation between donors and recipients. **(a)** The expression levels of NKG2D (*n* = 15), PD‐1 (*n* = 14), HLA‐DR (*n* = 15) and CD39 (*n* = 15) on the Vδ1 subset, as well as **(b)** NKG2D (*n* = 14), PD‐1 (n = 15), HLA‐DR (*n* = 14) and CD39 (*n* = 14) on the Vδ2 subset. The significant differences were determined by the nonparametric Wilcoxon test. *P*‐value levels are presented as *P < 0.05.

Recipients showed higher frequencies of NKG2D^+^ (median 79.55%, IQR_25‐75_ 54.33–95.15%), PD‐1^+^ (median 0.6%, IQR_25‐75_ 0.37–1.74%), HLA‐DR^+^ (median 15.55%; IQR_25‐75_ 10.88–23.78%) and CD39^+^ (median 1.5%, IQR_25‐75_ 0.68–4.77%) in Vδ2 T cells compared to donors (median 65.05%, IQR_25‐75_ 29.45–72.93%; median 0.11%, IQR_25‐75_ 0–1.40%; median 10.80%; IQR_25‐75_ 6.05–17.23%; median 1.0%; IQR_25‐75_ 0.64–1.27%, respectively), but no significant differences in the Vδ1 subset (Figure 2a and b). Chemokine receptors, exhaustion, tissue‐resident and activation markers were similarly expressed in Vδ1 and Vδ2 T cells between groups (Supplementary figure 3e and f). Overall, long‐term post‐aHCT recipients had a similar γδ T cell phenotype to their donors.

### TRG composition is restored in long‐term aHCT survivors

**Figure 3:**
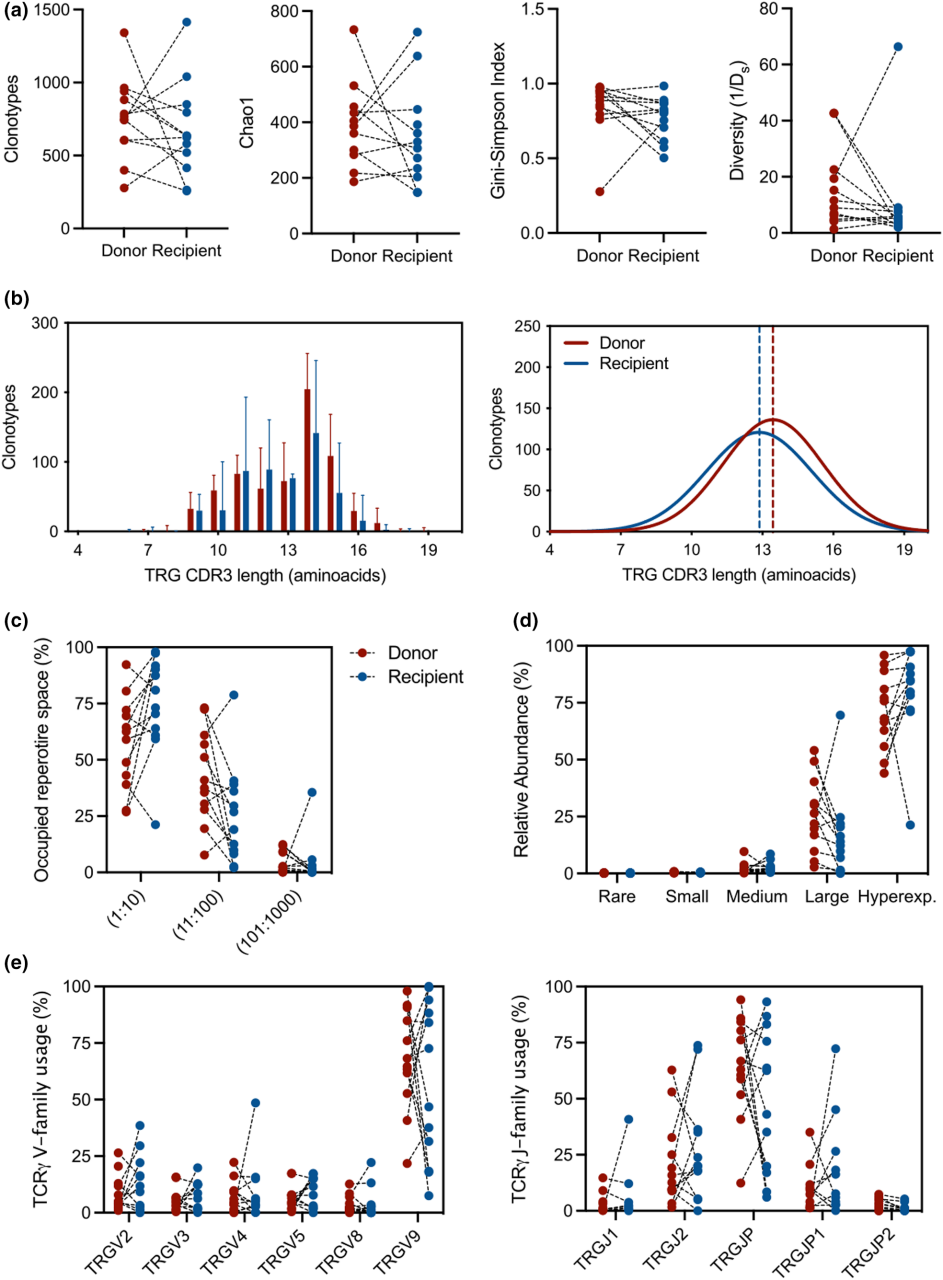
TRG diversity, distribution and clonality do not differ between donor and long‐term HSCT recipients. **(a)** Repertoire diversity calculation by the number of clonotypes, Chao1 estimator, Gini–Simpson and inverse Simpson's D indexes. **(b)** Distribution of unique CDR3 sequence lengths (left panel). Bars represent the median and interquartile range of 12 paired donor‐recipient subjects per group. Line graphs represent the nonlinear curve fitting (Gauss function) the patterns of unique CDR3 TRG lengths in each group (right panel). The vertical dashed lines indicate the group median CDR3 length. **(c)** TRG clonal proportion taken by the top *n* clonotypes divided into relative abundance of index intervals. **(d)** Homeostatic space proportions of clonotypes classified as hyperexpanded (> 1%), large (0.1–1%), medium (0.01–0.1%), small (0.001–0.01%) and rare (0–0.001%). **(e)** Individual TRG variable (V) and joining (J) gene segments usage frequency in the different sample groups. *n* = 12 paired donor‐recipient subjects.

A previous report has depicted the disturbed TRG composition early after aHCT when compared to baseline.^20^ In this study, the overall TRG diversity was assessed by the number of clonotypes, Chao1 estimator, Gini‐Simpson's and inverse Simpson's indices, with no differences observed between donor‐recipient pairs in the long term (Figure 3a). The TCR spectratype of CDR3 distributions also showed no differences between the groups, which presented mostly 14 amino acid long sequences and similar gaussian distributions (Figure 3b).

Next, we evaluated the occupied repertoire space by the top 10 (1:10), 100 (11:100) and 1000 (101:1000) clones, with no differences observed between the groups (Figure 3c). The relative abundance of clones classified as medium (0.01–0.1% of the repertoire), large (0.1–1%) or hyperexpanded (> 1%) also presented no differences between donor–recipient pairs (Figure 3d). Of note, no rare (0–0.001%) or small (0.001–0.01%) clones were found in any sample, supporting the oligoclonal nature of γδ T cell repertoire observed in both recipient and donor groups. Yet, the sequencing technical limitations may also prevent the detection of small clonotypes.

γδ T cells from aHCT grafts mostly express TRGV9 and TRGJP segments,^30^ matching the overall TRG gene segment distribution found in the peripheral blood. Both segments are also predominant in the early periods after aHCT, from 1 up to 6 months after HCT.^20^ When comparing long‐term recipients after aHCT with their matched donor pairs, we found no differences in variable nor joining segment usage (Figure 3e). Both groups presented a predominant use of TRGV9 and TRGJP gene segments, indicating a consistent similarity in the diversity and composition of γδ T cells between matched donors and recipients in the long term after aHCT.

### Overrepresented donor‐derived TRG clonotype expansion is associated with cGVHD severity

**Figure 4:**
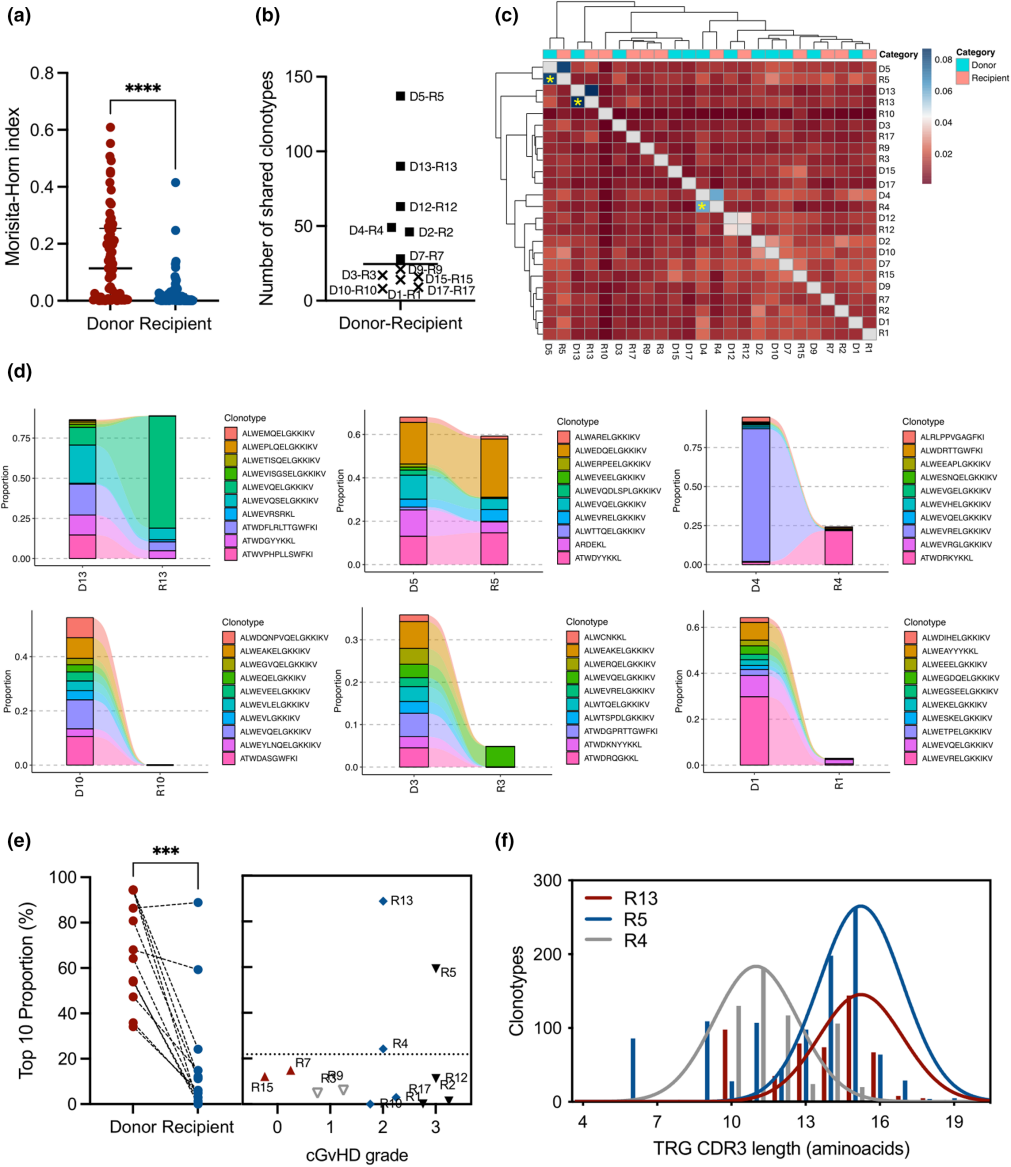
Patients who developed severe cGVHD present a higher persistence of donor‐derived private TRG clonotypes. **(a)** TRG repertoire overlap by Morisita‐Horn Index. Individual dots represent a pairing between two samples from the same group (total of 66 pairs). The index ranges from 0 (more private repertoires) to 1 (public repertoires, higher clonotype sharing between samples). All samples from the same group were overlapped with each other. ****P < 0.0001, Wilcoxon signed‐rank test. **(b)** Number of clonotypes shared by donor‐recipient pair (*n* = 12). **(c)** Heatmap and hierarchical clustering of samples based on Jaccard indices. Axis labels indicate donor and recipient numbers. Top *x*‐axis colour bars indicate sample groups. Samples presenting a high percentage of TRG repertoire similarity out of shared clonotypes are highlighted with a yellow asterisk (R5, R13 and R4). **(d)** Tracking of the donors' top 10 clonotypes in the recipients at long‐term after HSCT. Top panels depict recipients who developed moderate/severe cGVHD; lower panels depict recipients who developed mild cGVHD. Clonotypes are coloured randomly and do not match across different samples. **(e)** Left panel: Proportion of the TRG repertoire taken by the top 10 clonotypes in the donors and matched recipients. Right panel: Individual recipient top 10 proportion based on cGVHD grade. ***P < 0.001, the Wilcoxon signed‐rank test. **(f)** Distribution of unique CDR3 sequence lengths in the recipients who developed severe cGVHD and presented high levels of donor‐derived clonotype frequency.

Considering that the recipients' immune repertoire after aHCT is a chimera of cells from both donor (majority) and recipient (minority),^31^ we focused on understanding the clonotype sharing and presence of public clones in the donor–recipient pairs. First, we assessed the TRG repertoire similarity by calculating the Morisita–Horn (M‐H) index.^32^ We observed that the recipients displayed a significant presence of private clones (median M‐H: 0.114) when compared to matched donors (median M‐H: 0.005), who displayed a higher presence of public clonotypes (Figure 4a). This indicates that few clones would be shared between the donor–recipient pairs as a result of *de novo* generation of cell specificities after aHCT through thymic output of naïve γδ T cells.^20^ In fact, very few clones (median of 24 clones) were observed to be shared between the donor–recipient pairs in the long‐term after aHCT (Figure 4b), further confirming the high presence of private clones in the recipients.

To assess the public clone's sharing across the different donor and recipient pairs, we clustered all samples based on the Jaccard index.^32^ We found an overall low overlap between all samples, with few donor‐recipient pairs being clustered (Figure 4c). Three samples (R5, R13 and R4) showed a clear clustering between donor‐recipient pairs and presented the highest index among all samples, indicating a high degree of clonotyping shared between the samples (Figure 4c).

To identify possible clinically significant clonotypes, we tracked the top 10 donor‐derived clonotypes after aHCT and calculated their proportion in the TRG repertoire. For some samples, the top 10 most abundant clonotypes extracted from donor‐recipient pairs were still presented in a high frequency at the long term after aHCT, representing more than 80% of the entire TRG diversity (R13), while for others the top 10 clonotype proportion reduced from above 40% to undetectable levels (R10, Figure 4d). This reduction was significant for most of the samples analysed (Figure 4e), further confirming the private distribution of the recipients' repertoire after aHCT.

Alloreactive donor‐derived αβ T cells are key players in the development of acute and chronic GVHD,^33^, ^34^, ^35^ while the role of γδ T cells is still unclear because of their MHC‐independent response mechanisms.^36^ We investigated the proportion of donor‐derived clonotypes to determine their link to cGVHD in recipients and hypothesised that specific TRG clones could be associated with cGVHD onset. The top 10 clonotype proportions accounted for a median of 8.6% of all TRG repertoires, with the 25th and 75th quartiles of 1.73% and 21.85%, respectively. Notably, three recipients (R5, R13 and R4) who developed severe cGVHD (grades 2 or 3) exhibited substantially higher proportions of 59.25%, 88.85% and 24.17%, respectively (Figure 4e). Further examination revealed clonotypes in severe cGVHD donor‐recipient pairs that expanded over 1.5‐fold following aHCT. These expanded clonotypes may suggest a possible role of γδ T cells in cGVHD development (Supplementary figure 4). Additionally, the CDR3 spectratype analysis showed distinct sequence distributions between samples, indicating their unique, private distribution patterns (Figure 4f). This reinforces the potential association between specific γδ T cell clonotypes and the development of severe cGVHD. However, it is not excluded that their expansion could also be attributed to cell reconstitution processes independent of cGVHD.

### HLA‐DR^+^ Vδ1 T cells are associated with ongoing moderate/severe cGVHD

**Figure 5:**
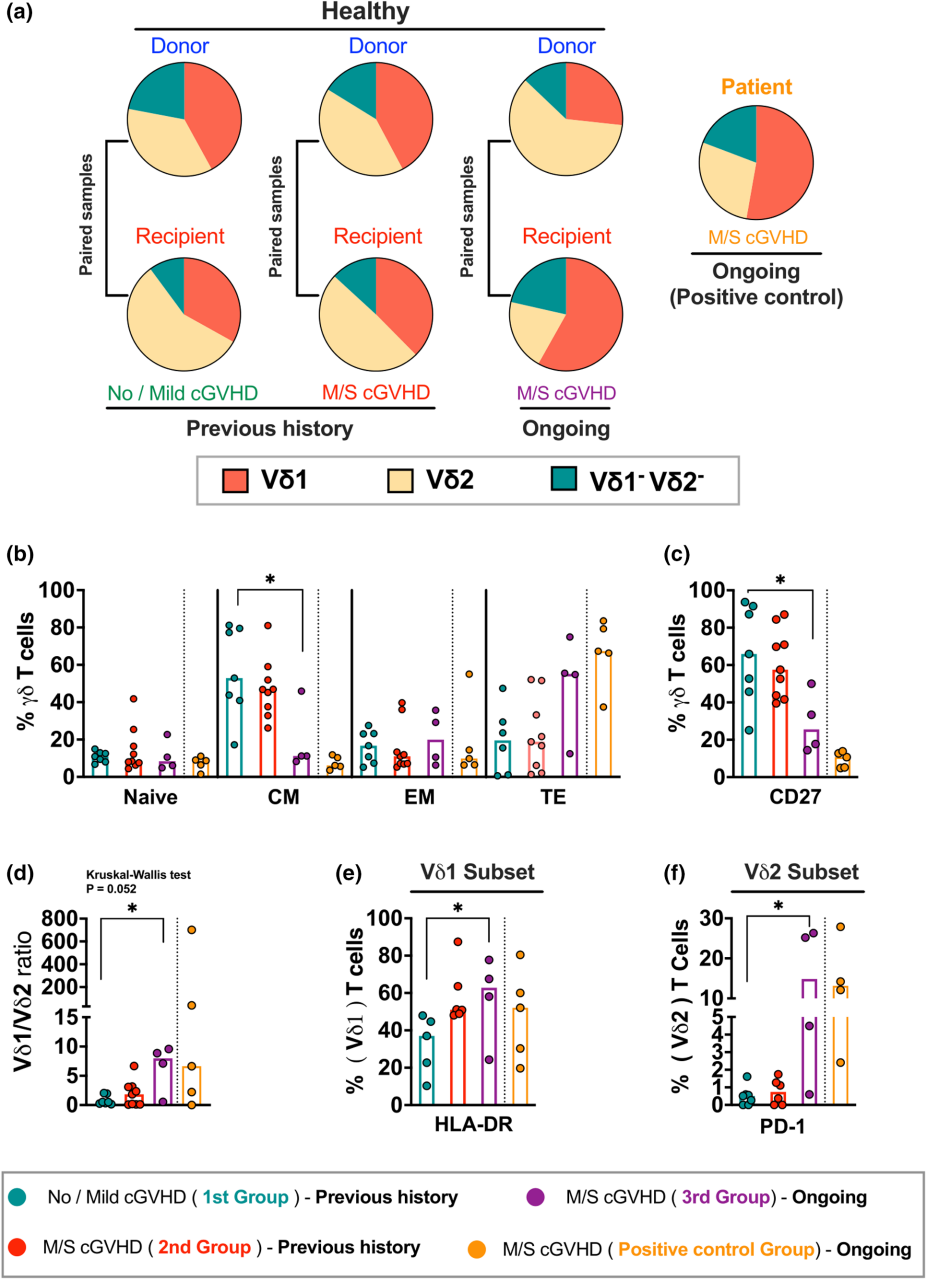
The association of γδ T cells and their subsets with cGVHD in recipients. **(a)** The proportion of the γδ T cell subset (Vδ1, Vδ2 and Vδ1^−^Vδ2^−^ subsets) distribution was examined between recipients and corresponding donors based on cGVHD severity and status: previous history of no/mild cGVHD recipients and matched donors (*n* = 7), previous history of M/S cGVHD recipients and matched donors (*n* = 9), ongoing M/S cGVHD recipients and matched donors (*n* = 4) as well as an additional cohort of ongoing M/S cGVHD patients as a positive control (*n* = 5). **(b–f)** The analysis was performed based on cGVHD at the time of sampling. Significances are provided for comparing ongoing M/S cGVHD (3rd group) and previous history M/S cGVHD (2nd group) to previous history no/mild cGVHD (1st group). **(b)** The frequency of memory phenotype (CD27 & CD45RO) of total γδ T cells, **(c)** the frequency of CD27^+^ γδ T cells, **(d)** the ratio of Vδ1/ Vδ2 between 1st group (*n* = 7), 2nd group (*n* = 9), 3rd group (*n* = 4) and positive control group (*n* = 5), **(e)** differences in HLA‐DR expression on Vδ1 T cells between 1st group (*n* = 5), 2nd group (*n* = 6), 3rd group (*n* = 4) and positive control group (*n* = 5) and **(f)** differences in PD‐1 expression on the Vδ2 subset between 1st group (*n* = 7), 2nd group (*n* = 6), 3rd group (*n* = 4), and positive control group (*n* = 4). Bars indicate median values. The *P*‐value was obtained by the Kruskal–Wallis test followed by Dunn's multiple comparisons test. *P*‐value levels are presented as *P < 0.05. M/S, Moderate/Severe.

Our investigation extended beyond the association between acute and chronic GVHD and γδ T cell reconstitution. Given that cGVHD is a late multi‐organ disorder complication, and having observed a potential association with donor‐derived clonotypes, and because of a limited sample size obtained from long‐term aHCT survivors who had an ongoing moderate/severe (M/S) cGVHD at the time of sampling (*n* = 4; R2, R4, R5 and R12), a supplementary cohort of patients with M/S cGVHD was included as a positive control (*n* = 5). So, we first analysed the distribution of γδ T cell subsets among cGVHD recipients and their corresponding donors (Figure 5a), with the Vδ1, Vδ2, Vδ1^−^Vδ2^−^ proportions being similar among donors and recipients of the no/mild and past M/S cGVHD. Interestingly, we observed a trend towards a higher proportion of the Vδ1 subset (mean ± SEM: 56.4 ± 10.63%) and Vδ1^−^Vδ2^−^ subsets (mean ± SEM: 20.8 ± 7.52%) in recipients with ongoing M/S cGVHD compared to their matched donors (mean ± SEM: 25.9 ± 6.88% & 12.5 ± 4.08%, respectively) who exhibited a more Vδ2 proportion (mean ± SEM: 58.6 ± 8.98%), although this was not statistically significant. Likewise, the positive control group with M/D cGVHD displayed a higher proportion of the Vδ1 subset (mean ± SEM: 49.9 ± 15.4%) than the Vδ2 subset (mean ± SEM: 26.4 ± 18.4%).

**Figure 6:**
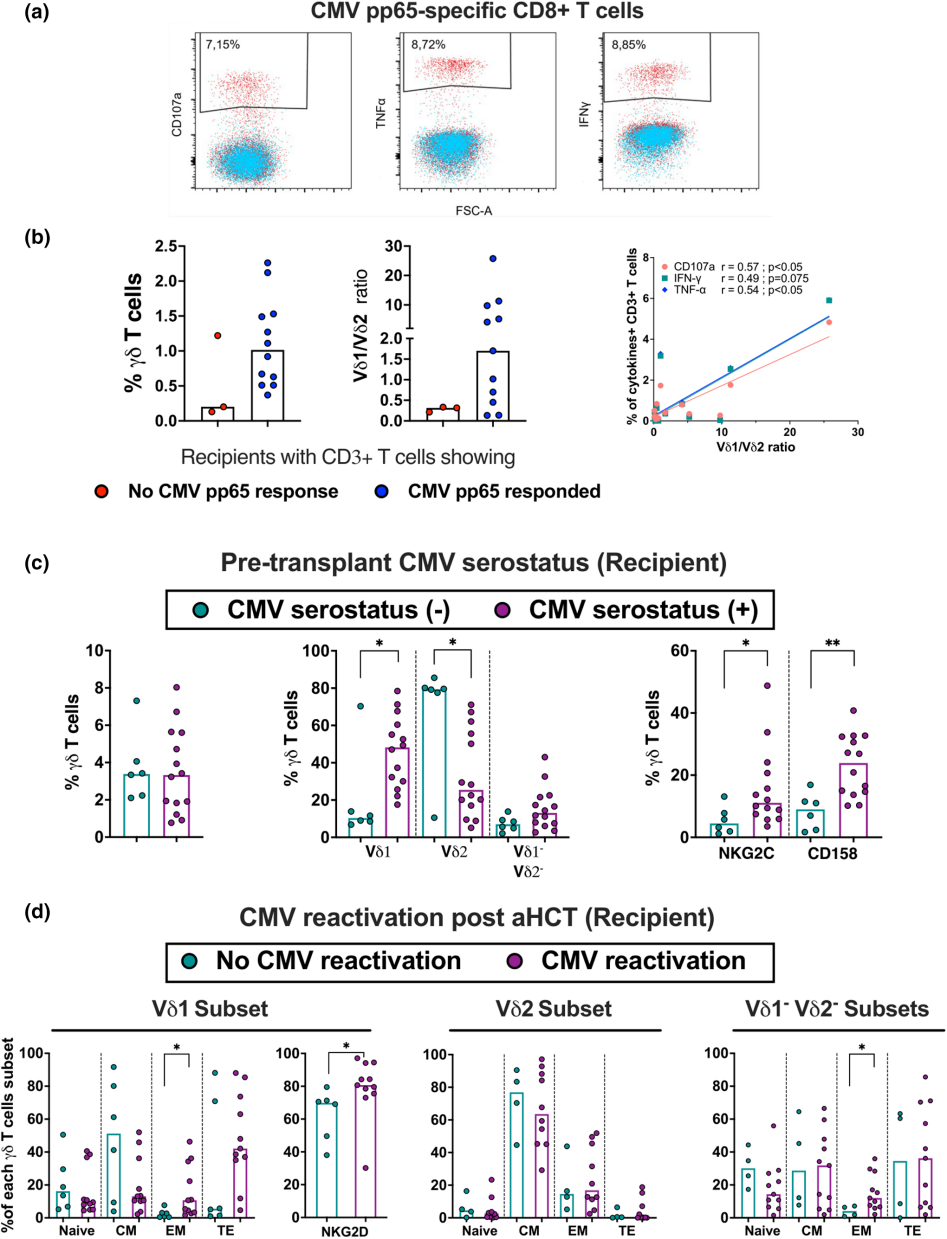
Lasting imprint on recipient γδ T cells with pre‐transplant CMV serostatus and CMV reactivation post aHCT. **(a)** Representative example of the FACS plot showing the CMV‐specific CD8 T cell response to CMVpp65 stimulation. **(b)** Frequencies of γδ T cell and Vδ1/ Vδ2 ratio in recipients defined based on CD3^+^ T cell reactivity (blue dots) or no reactivity (red dots) to CMV pp65 peptide stimulation, and correlation analysis between cytokine‐producing CD3^+^ T cells in response to CMV pp65 stimulation and Vδ1/ Vδ2 ratio In total γδ T cell: negative (*n* = 3) and positive (*n* = 12), and Vδ1/Vδ2 ratio: negative (*n* = 3) and positive (*n* = 11). **(c)** Frequencies of γδ T cells, Vδ1, Vδ2 and Vδ1^−^ Vδ2^−^subsets and expression of NKG2C and CD158 among γδ T cells based on pre‐transplant CMV serostatus (−) (*n* = 6) and CMV serostatus (+) (*n* = 14) of recipients. **(d)** Memory phenotype of Vδ1, Vδ2 and Vδ1^−^ Vδ2^−^ subsets out of γδ T cells and expression of NKG2D among Vδ1 γδ T cells based on CMV reactivation. In the Vδ1 subset: No CMV reactivation (*n* = 6) and CMV reactivation (*n* = 11). In the Vδ2 subset: No CMV reactivation (*n* = 4) and CMV reactivation (*n* = 10). In Vδ1^−^ Vδ2^−^ subsets: No CMV reactivation (*n* = 4) and CMV reactivation (*n* = 11). Bars indicate median values. Significant differences were calculated by the Mann–Whitney test and correlations by Spearman's test. *P*‐value levels are presented as *P < 0.05 and **P < 0.01.

We then further divided recipients into three groups: past no/mild cGVHD (first group, *n* = 7), past M/S cGVHD (second group, *n* = 9) and ongoing M/S cGVHD at the time of sampling (third group, *n* = 4) (Figure 5b–f). The proportion of central memory (CM) γδ T cells expressing CD27 was significantly reduced in the ongoing M/S cGVHD group compared with the no/mild group (Figure 5b and c). A similar reduction was observed in the positive control group. Conversely, there was a trend towards an increase in terminally differentiated (TE) γδ T cells in the ongoing M/S cGVHD group compared to the other groups, although it was not statistically significant. The positive control group again showed a similar trend with TE γδ T cells (Figure 5b). Further analysis showed that recipients with ongoing M/S cGVHD presented a higher Vδ1/Vδ2 ratio compared to the no/mild cGVHD group, which was also observed in the positive control group (Figure 5d). Recipients in the ongoing M/S cGVHD group exhibited higher HLA‐DR expression in Vδ1 T cells and higher PD‐1 expression in Vδ2 T cells compared to the no/mild group; a similar increase was also observed in the positive control group (Figure 5e and f). Additionally, the frequency of CCR7^+^ Vδ1 T cells and CCR6^+^ Vδ2 T cells was significantly lower in recipients with M/S cGVHD compared to those with no/mild cGVHD. This reduction in CCR7 and CCR6 was also observed in the positive control group with M/S cGVHD (Supplementary figure 5a and b). Together, these results revealed that cGVHD has a significant impact on γδ T cell functionality long‐term after aHCT, causing phenotypic alterations, particularly in the Vδ1^+^ T cell subset. An elevated frequency of HLA‐DR Vδ1 T cells (Figure 6e, Supplementary figure 5a) might be associated with the severity of cGVHD, suggesting it can be a target for therapeutic intervention.

Next, we compared the γδ T cell phenotypes between recipients with aGVHD grades 0‐I and II‐III (Supplementary figure 6). Recipients with grades II‐III aGVHD had a significantly lower proportion of PDL1‐positive cells among γδ T cells and the Vδ2 subset than those with grades 0‐I (Supplementary figure 6a and c). The proportion of the effector memory subset within the Vδ2 γδ T cells was significantly higher in recipients with grades II‐III aGVHD than in grades 0‐I, whereas there were no significant differences in the memory phenotype in total γδ T cells and the Vδ1 subset (Supplementary figure 6a–c). Conversely, recipients with grades II‐III aGVHD had significantly lower CD27 expression on the Vδ2 subset (Supplementary figure 6c). Additionally, these recipients showed decreased CD69 and CD103 expression and increased DNAM‐1 expression in the Vδ2 subset compared to those with grades 0–I (Supplementary figure 6c). These findings suggest that phenotypic differences, particularly in the Vδ2 subset, can be observed between recipients with grades 0–I and II–III. However, these differences may also arise from other unknown factors occurring in the meantime.

We then examined the association between γδ T cell functionality on acute and chronic GVHD (Supplementary figure 7). After mitogenic (PMA/ionomycin) stimulation, recipients with grade II‐III aGVHD and M/S cGVHD showed a higher proportion of MIP‐1β expressing‐γδ T cells (Supplementary figure 7a and b). Specifically, an increased proportion of MIP‐1β‐expressing Vδ1 and Vδ2 γδ T cells was seen in recipients with grades II‐III aGVHD, with a similar increase noted in Vδ2 γδ T cells in those with M/S cGVHD (Supplementary figure 7a and b). Additionally, Vδ2 γδ T cells from grade II‐III aGVHD recipients showed a higher proportion of IFN‐γ and TNF‐α, but cGVHD severity groups did not differ significantly (Supplementary figure 7a and b). Overall, the elevated levels of MIP‐1β were associated with recipients who experienced GVHD severity in acute and chronic.

### Vδ1 γδ T cells are associated with long‐term CMV control after aHCT

To investigate the relationship between CMV response and γδ T cell frequency and composition, we analysed (i) recipients' T cells reactivity to CMV pp65 peptide stimulation, (ii) recipients' pre‐transplant CMV serostatus and (iii) the history of CMV reactivation post aHCT. The T cell reactivity to CMVpp65 peptide stimulation was investigated through degranulation (CD107a), TNF‐α, and IFN‐γ expression (Figure 6a). Recipients γδ T cells were divided into responder (*n* = 12) and non‐responder (*n* = 3) based on their T cell response to CMV stimulation (Figure 6b). The responder group had a trend towards a higher frequency of γδ T cells and Vδ1/ Vδ2 ratio compared to non‐responders. Furthermore, the frequency CD107a,^+^ IFN‐γ^+^ and TNFα^+^ T cells correlated with Vδ1/ Vδ2 ratio, suggesting an association of the CMVpp65‐specific T cells response with Vδ1 T cells. Similarly, at the recipients' CMV serostatus level, while no significant difference in total γδ T cells between groups was observed, CMVsero^+^ recipients had a higher Vδ1 subset proportion (Figure 6c). CMVsero+ recipients also had more γδ T cells expressing NKG2C and CD158. Post‐aHCT, recipients with CMV reactivation (median 55 days post‐aHCT) showed a higher proportion of effector memory in Vδ1^+^ and Vδ1^−^Vδ2^−^ subsets (Figure 6d), and higher NKG2D expression among Vδ1 T cells compared to those without reactivation. No significant difference was observed in the Vδ2 T cell memory profile between groups. These findings suggest that the association between CMV serostatus, reactivation and the proportion of Vδ1^+^ and Vδ1^−^Vδ2^−^ T cells supports their role in long‐term CMV control.

Next, we assessed the function of recipients’ γδ T cells and their subsets (Supplementary figure 8a–c). Upon mitogenic (PMA/ionomycin) stimulation, Vδ2^+^ γδ T cells exhibited higher NKG2D expression but reduced production of MIP‐1β as compared to Vδ1^+^ γδ T cells (Supplementary figure 8b), indicating long‐term aHCT γδ T cells remain functional. Additionally, NKG2D^+^ γδ T cells showed a higher production of IFN‐γ and TNF‐α compared to NKG2D^−^ γδ T cells (Supplementary figure 8c), which are attributable to the innate‐like phenotype.

## Discussion

In aHCT, the early reconstitution of γδ T cells plays a crucial role in the control of opportunistic infections, tumor surveillance and exhibits a direct correlation with increased patient survival rates.^22^, ^25^, ^29^ Despite several studies highlighting the significance of γδ T cells and their specific subsets in the graft, as well as the association between their enhanced reconstitution and favorable clinical outcomes, a notable gap exists in understanding γδ T cells' long‐term homeostatic steady state after aHCT.^23^, ^26^, ^37^, ^38^, ^39^ To our knowledge, we provide for the first‐time an in‐depth analysis between the association of γδ T cells' phenotype, immune repertoire and functionality of ≥ 8 years post aHCT long‐term survivors with clinical complications.

In contrast to αβ T cells, the γδ T cell profile in long‐term survivors demonstrated remarkable comparability to their respective donors with regard to naïve/memory profiles and TCR diversity/composition. The rapid γδ T cell reconstitution post‐HCT has been associated with the peripheral expansion of donor‐derived T cells present in the graft.^20^, ^25^ Previous studies have shown a positive correlation between the early recovery of γδ T cells in HCT recipients and donor γδ T cell contents,^40^ and γδ T cells have even been shown to be the predominant T cell population in paediatric HCT during the first week's post‐transplantation.^41^ Interestingly, only Vδ2 T cells presented a higher degree of activation by the surface expression of NKG2D, CD39, HLA‐DR and PD‐1 compared to donors. This activated phenotype could be attributed to the quick response to microbe‐associated antigens with innate‐like activation driven by the NKG2D receptor,^42^ especially on PD‐1‐expressing γδ T cells.^43^ Additionally, Vδ2 T cells represent the primary γδ T cell subset in the peripheral blood and are under investigation as cellular immunotherapy for cancer treatment because of their activation potential when encountering tumors.^44^ A recent study observed that Vδ2^+^ CD26^+^ T cells produced a high level of cytokines early after aHCT in patients compared to healthy controls, which was associated with favorable outcomes and a lower risk of relapse.^45^ While Vδ1 T cells are predominantly tissue‐resident and undergo a process of adaptive‐like immune responses upon antigen exposure.^46^, ^47^

CMVsero+ donors exhibited a higher frequency of Vδ2^−^ T cells and were mainly effector/memory cells, also expressing a high proportion of NKG2C and CD158 compared to CMVsero‐ donors. Moreover, the patients transplanted with a CMV+ graft (D+/R−) who were experiencing CMV infection showed a faster expansion of Vδ2^−^ T cells than patients with (D−/R+) who did not, indicating these expanded cells act as memory cells with a rapid response.^48^ The NKG2C acts as an activating receptor that recognises the CMV‐derived UL40 peptide presented by the HLA‐E molecule.^49^ In addition, a higher frequency of effector memory Vδ2^−^ γδ T cells (Vδ1^+^ and non Vδ1/Vδ2) was also found in CMV‐experienced reactivation recipients. The Vγ9^−^Vδ2^+^ and Vδ2^−^ γδ T cells, especially Vδ1^+^ T cells, are known to be crucial during CMV infection/reactivation by exhibiting adaptive‐like properties for CMV control,^48^, ^50^, ^51^, ^52^ suggesting that these cells play a key role in the long‐term control of CMV.

The TCR repertoire composition following aHCT is associated with several complications, and a skewed γδ T cell repertoire with overrepresented specific clones has been observed after viral reactivation, notably CMV.^20^, ^30^, ^53^ TCR repertoire diversity characteristics post‐aHCT offer valuable insights into the immune reconstitution dynamics,^54^ but most studies examining γδ T cell repertoires have focused on short‐term outcomes after transplantation, with limited research into the role of the thymus in reconstituting γδ T cells in long‐term survivors. We found that the proportion of naïve γδ T cells and the composition of the TRG repertoires were similar between donors and recipients. This qualitative resemblance in the diversity and CDR3 sequence structure suggests that the γδ T cell repertoire normalises over time, leading to a restored TRG composition and stable γδ T cell homeostasis in long‐term survivors post‐HCT. Furthermore, the presence of distinct and private clonotypes in the recipients' γδ TCR repertoire suggests a potential manifestation of *de novo* generation of naïve γδ T cells through thymic output.

The monitoring of TCR repertoire changes over time post‐HSCT has been used to understand the role of αβ T cells in GVHD development, with both higher^55^, ^56^ and lower^54^, ^57^ TCR diversities being reported. We observed that 3 recipients who developed severe cGVHD showed a high frequency of donor‐derived clonotypes even in the long term after aHCT at a median of 12 years 12 (range 9–14 years). Similar to our observation, a recent study found that the association of the skewed γδ T cell clonality with grade 3 of aGVHD after UCB transplantation in children,^58^ indicating a possible association between γδ T cells and cGVHD development. Interestingly, we also observed an inversion of the Vδ1/Vδ2 cells ratio in cGVHD patients. The Vδ1 subset, significantly less frequent in adult blood circulation, was surprisingly more prevalent than the Vδ2 subset in patients who have developed or have ongoing M/S cGVHD when compared to matched donors. Additionally, we found elevated expression of HLA‐DR in Vδ1 T cells within the M/S cGVHD patients, indicating the high activation status of these cells and a possible association with GVHD development.^59^, ^60^

Our study presented some limitations. This included a small sample size and highly heterogeneous cohorts regarding the conditioning regimen, product and GVHD prophylaxis, which may have affected the generalisability of our results. Additionally, providing absolute counts for the γδ T cell compartment is challenging because the samples were collected ≥ 8 years post‐transplantation, and there are potential variations in blood volume across samples. Finally, we were unable to perform a separate repertoire analysis of Vδ1 and Vδ2 subsets, which remain an important step to enhance the interpretation of clonality and diversity in our analysis. Therefore, further longitudinal studies with a larger cohort of patients and donor graft samples are needed to strengthen the findings.

### Conclusions

Our data show that the reconstitution of γδ T cells appears to achieve homeostasis with a normalised immune repertoire, high functional capacity and CMV control after long‐term aHCT. However, cGVHD represents the most significant influence on thymic‐dependent reconstitution, potentially leading to donors' overrepresented peripheral clones for reconstitution. We also observed an inversion of the Vδ1/Vδ2 cells ratio by enriching the Vδ1^+^ subset and an increased frequency of HLA‐DR^+^ Vδ1^+^ T cells in ongoing M/S cGVHD recipients, suggesting a possible association between Vδ1^+^ subset and cGVHD development. These results are interesting for future research to gain insight into the Vδ1‐chain repertoire in GVHD patients.

## Methods

### Study samples and design

Long‐term survivors who underwent aHCT at the Center for Cell Therapy and Allogeneic Stem Cell Transplantation, Karolinska University Hospital, Huddinge, Sweden, between 1983 and 2005, along with their corresponding sibling donors, were invited to participate in a long‐term follow‐up. Exclusion criteria included re‐aHCT and unmatched sibling pairs. Peripheral blood samples were collected from recipients and their donors (*n* = 20) at least 8 years post‐aHCT, between 2008 and 2014. Detailed patient characteristics and sampling times are provided in Table 1 and Supplementary figure 1, respectively. The study was approved by the regional ethics review board at Karolinska Institutet and conducted in accordance with the Declaration of Helsinki. All participants provided signed consent forms. A supplementary cohort of patients with moderate/severe graft‐versus‐host disease (GVHD) (*n* = 5) was included as a positive control (Supplementary table 1).

### Sample preparation

Peripheral blood mononuclear cells (PBMCs) were isolated by density gradient separation using Lymphoprep (Fresenius Kabi, Oslo, Norway) as specified previously^61^ and cryopreserved until use in RPMI‐1640 media (HyClone; Thermo Fisher Scientific, Waltham, MA, USA) supplemented with 10% human AB serum (Karolinska University Hospital, Huddinge, Sweden), 1% penicillin/streptomycin (P/S; Life Technologies, Paisley, UK) and 10% dimethyl sulfoxide (DMSO, Sigma‐Aldrich, Saint Louis, MO, USA). The frozen cells were stored in liquid nitrogen (−196°C) at the Clinical Immunology laboratory at Karolinska University Hospital in Huddinge.

### Immunophenotyping

Frozen PBMCs were thawed, washed and resuspended in PBS. Extracellular staining was performed as described^62^ and cell viability was assessed using 7AAD (BD Biosciences, Milpitas, CA, USA). Acquisition was performed using the CytoFLEX cytometer (Beckman Coulter, Brea, CA, USA). All the antibodies used are provided in Supplementary table 2. Acquired FACS data was analysed using FlowJo V10 software (BD Bioscience, Ashland, OR, USA). Gating was determined using fluorescence minus one (FMO) controls. Gating strategy is shown in Supplementary figure 2. A minimum of 75 absolute events was used as a threshold to report subpopulations for analysis.

### TRG next‐generation sequencing

Immunosequencing of the CDR3 region of TRG was performed by iRepertoire (Huntsville, AL, USA). In brief, γδ T cells were sorted using the Sony MA900 (Sony Biotechnologies, San Jose, CA, USA), with a median sorting efficiency of 92%. RNA was immediately isolated using the RNeasy Micro kit (Qiagen, Hilden, Germany) according to the manufacturer's instructions and stored at −80°C. NanoDrop 2000 (Thermo Fisher Scientific, Wilmington, Delaware, USA) was used to determine RNA concentration and purity. Then, RNA was used as the template of reverse transcription PCR using multiplexed V and J region specific primers to amplify TCR γ‐chain CDR3 rearrangements. Sequence reads were aligned to the IMGT database to map productive clonotypes with full amino acid and gene segment usage.

### Analysis of NGS data

Sequencing data was initially analysed using the iRweb software (iRepertoire). Raw data was downloaded and further analysed using the Immunarch R package (https://github.com/immunomind/immunarch) to assess TRG CDR3 diversity, clonal space homeostasis and proportion, spectratype and V−/J‐segment usage. Only productive CDR3 rearrangements were included in the analysis. Data were normalised by downsampling the repertoires to the size of the smallest dataset to allow unbiased comparison between samples. Repertoire similarities were calculated by using the Morisita‐Horn and Jaccard Indices. Recipient clonotypes with a fold increase higher than 1.5 as compared to the donor were extracted, aligned using Clustal Omega (https://www.ebi.ac.uk/Tools/msa/clustalo/) and motif sequence logos of the amino acid CDR3 sequences were generated on the WebLogo server (http://weblogo.threeplusone.com/create.cgi).

### γδ T cells functional assay

After thawing, PBMCs of recipients were incubated in RPMI‐1640 medium supplemented with 10% fetal bovine serum (FBS; HyClone, Pasching, Austria) and 1% P/S (Life Technologies, Paisley, UK) overnight for resting. Subsequently, cells were either subjected to 6‐h stimulation with PMA and ionomycin (25 ng mL^−1^ and 1 mg mL^−1^, respectively, Sigma‐Aldrich) or PepTivator CMV pp65 –premium grade (1 μg pept mL^−1^, Miltenyi Biotec, Bergisch Gladbach, Germany). Stimulation was assessed in the presence of 10 mg/mL Brefeldin A (Sigma‐Aldrich), 0.7 μL of Golgi Stop (monensin, BD Biosciences, San Diego, CA, USA) and PE anti‐CD107a (BD Biosciences). Then, stimulated cells were washed with PBS followed by staining with the LIVE/DEAD Fixable Aqua Dead Cell Stain Kit (Thermo Fischer Scientific, Eugene, OR, USA) after CMV pp65 stimulation or Fixable Viability Stain 780 (BD Biosciences) after PMA/ionomycin stimulation, according to the manufacturer's instructions. After washing, extracellular staining (Supplementary table 2) was performed for 15 min followed by fixation and permeabilisation (BD Biosciences). Fixed cells were then stained with intracellular antibodies (Supplementary table 2) for 30 min. Cells were acquired using the CytoFLEX cytometer (Beckman Coulter).

### Statistical analysis

Data were analysed using GraphPad Prism 10.2.0 software. Differences between cell subset frequency and expression of specific markers of donor‐recipient paired samples were done using the Wilcoxon matched‐pairs signed rank test. Differences between 2 or more groups of unpaired samples (GVHD, CMV status/reactivation) were analysed using the Mann–Whitney *U‐*test or Kruskal–Wallis followed by Dunn's multiple comparisons test. Correlation analysis was performed using Spearman's test. The median is represented in the graphical representations. The significance threshold was set at 0.05.

## Author contributions

**Faisal Alagrafi:** Data curation; formal analysis; methodology. **Arwen Stikvoort:** Conceptualization; data curation; methodology. **Ahmed Gaballa:** Conceptualization; data curation; investigation. **Martin Solders:** Data curation; resources. **Olle Ringden:** Investigation; methodology; resources. **Thomas Poiret:** Conceptualization; data curation; formal analysis; resources. **Lucas CM Arruda:** Conceptualization; methodology; formal analysis. **Michael Uhlin:** Resources; supervision.

## Conflict of interest

The authors declare no conflict of interest.

## Supporting information


Supporting Information

## Data Availability

Data are available upon request from the corresponding authors.
