# Supplementary material for: γδ T cell characterisation in the long term after haematopoietic stem cell transplantation and its impact on CMV control and cGVHD severity
Source: Clin Transl Immunology. 2025 Mar 7;14(3):e70027. doi: 10.1002/cti2.70027 (PMC11886888; doi:10.1002/cti2.70027)
Supplement: Supplementary file 1 — Supporting Information [file CTI2-14-e70027-s001.docx]

**Supporting Information**

**Supplementary table 1.** Clinical characteristics of a supplementary cohort of patients.

|  | **Patients** |
| --- | --- |
| **Total Number** | 5 |
| **Gender:** female/male, n | 1/4 |
| **Median age at time of HCT (range)** | 57 (29-67) |
| **CMV serostatus pre- HCT, n**  D+/R+  D-/R+ | 4  1 |
| **Underlying disease, n**  AML  MDS/MPS  CML  ST | 1  2  1  1 |
| **Conditioning intensity, n** |  |
| Myeloablative | 2 |
| Reduced intensity | 3 |
| **Graft source**, n |  |
| Bone marrow | 1 |
| PBSC | 4 |
| **CMV reactivation,** n | 5 |
| **aGVHD:** 0-I/II-III, n | 1/4 |
| **cGVHD:** no-mild/ moderate-severe, n | 0/5 |

AML = Acute myeloid leukemia, MDS = Myelodysplastic syndromes, MPS = Myeloproliferative syndrome, CML = Chronic myeloid leukemia, ST = solid tumor, PBSC = Peripheral blood stem cells, CMV = Cytomegalovirus, aGVHD = acute graft-versus-host disease, cGVHD = chronic graft-versus-host disease.

**Supplementary table 2.** Flow cytometry antibodies.

ECS = extracellular staining, ICS = intracellular staining.

**Supplementary figure 1.** Sampling time between donors and recipients after aHCT (n = 20).

**Supplementary figure 2.** Representative example of the gating strategy used for flow cytometry data and cell sorter. The gating strategy was evaluated based on singlet events and gated on lymphocytes (determined by size and granularity), living cells (identified by the absence of 7-AAD), CD3 (indicating T cells) and further gating to characterize the immune cell populations of interest. **(a)** Gating strategy of γδ T cells and their subsets, **(b)** αβ T cells (gated from γδ^-^) for phenotypic analysis. Sub-gating was done for at least 75 events for markers on γδ T subsets. **(c)** Representative gating plot for markers analysis. **(d)** Gating strategy of γδ T cells for sorting. Values in all plots indicates proportion of parent population.

**Supplementary figure 3.** Phenotypic characterization of γδ T cells and their subsets between donors and recipients. **(a)** Frequency of CD4^+^, CD8^+^, CD4^+^ CD8^+^, and CD4^-^ CD8^-^ on total γδ T cells (n = 20). **(b)** Frequency of NK cell receptors (CD56, DNAM-1, NKG2A, NKG2C, NKG2D, CD158, CD16, and NKP44), **(c)** Frequency of several chemokine receptors (CCR2, CCR5, CCR6, CCR7, CCR9, and CXCR3) and (**d**) expression of CD27, CD137, and PDL1 on total γδ T cells (n = 20). (e, f) Frequency of several chemokine receptors CCR2, CCR5, CCR6, CCR7, CCR9, CXCR3, and CX3CR1on both Vδ1 and Vδ2 subset (Vδ2: n = 14 ; Vδ1: n = 16), and frequency of CD69 (Vδ2: n = 14 ; Vδ1: n = 15), CD86 (Vδ2: n = 14 ; Vδ1: n = 15), TIM-3 (Vδ2: n = 15 ; Vδ1: n = 14), CD154 (Vδ2: n=14 ; Vδ1: n=15), and CD103 (Vδ2: n = 14 ; Vδ1: n = 15) within Vδ2 and Vδ1 subsets.

**Supplementary figure 4.** Proportion of expanded donor-derived clonotypes. (Left) Proportion of donor-derived clonotypes restricted to a donor-recipient pair that have expanded above 1.5-fold after aHCT. (Right) Amino acid enrichment analysis of the expanded clonotypes using the WebLogo application of the donor-recipient pair **(a)** R5, **(b)** R4 and **(c)** R13. The amino acids are coloured according to their physicochemical properties (black, hydrophobic; green, polar; red, acidic; blue, basic; neutral, purple).

**Supplementary figure 5.** The association of γδ T cells and their subsets with cGVHD in recipients. The analysis was conducted by comparing the cGVHD grade of the recipients in this study in two groups: previous history no/mild cGVHD (1^st^ group) versus previous history combined with ongoing M/S cGVHD (2^nd^ group). An additional cohort of ongoing M/S cGVHD was added as a positive control. (**a**) The differences in CCR7 expression of Vδ1 subset between 1^st^ (n = 6), 2^nd^ group (n = 11) and positive control (n = 5), and in HLA-DR expression of Vδ1 T cells between 1^st^ (n = 5), 2^nd^ group (n = 10) and positive control (n = 5). (**b**) The differences in CCR6 expression of Vδ2 subset between 1^st^ group (n = 7), 2^nd^ group (n = 9), and positive control (n = 4). Each bar chart shows median values. The *P*-value was calculated using the Mann-Whitney test. *P*-value levels are presented as **P* < 0.05 and ***P* < 0.01. M/S = Moderate/Severe.

**Supplementary figure 6.** The association between aGVHD and γδ T cell reconstitution. The analysis was performed based on aGVHD grade (0-I) and (II-III). **(a)** The frequency of memory phenotype (CD27 & CD45RO) of total γδ T cells and frequency of PDL1 γδ T cells were analysed between grade 0-I (n = 12) and grade II-III (n = 8) **(b)** The frequency of memory phenotype (CD27 & CD45RO) between grade 0-I (n = 10) and grade II-III (n = 7) on Vδ1 subset. **(c)** The frequency of memory phenotype (CD27 & CD45RO), frequency of CD27 between grade 0-I: (n = 7) and grade II-III (n = 7) and the frequency of PDL1 between grade 0-I (n = 8) and grade II-III (n = 7) on Vδ2 subset. The expression of DNAM-1 between grade 0-I (n = 7) and grade II-III (n = 7), expression of CD69 and expression of CD103 between grade 0-I (n = 9) and grade II-III (n = 7) on Vδ2 subset. Each bar chart is shown as median. The significant differences were compared using the Mann-Whitney test. *P*-value levels are presented as **P* < 0.05 and ***P* < 0.01. CM: central memory, EM: effector memory, and TE: terminally differentiated.

**Supplementary figure 7.** The association of functional data from γδ T cell to aGVHD and cGVHD. The analysis was performed based on grade (0-I) and (II-III) of aGVHD and (no/mild) and (M/S) of cGVHD. **(a)** Comparison of proportions of IFN-γ, TNF-α, MIP-1β-expression by γδ T cells, Vδ1 γδ T cells, and Vδ2 γδ T cells between grade 0-I (n = 9) and grade II-III (n = 6) of aGVHD. **(b)** Comparison of proportions of IFN-γ, TNF-α, MIP-1β-expression by γδ T cell, Vδ1 γδ T cell, and Vδ2 γδ T cell between no/mild (n = 4) and M/S (n = 11) of cGVHD. Each bar chart is shown as median. The significant differences were compared using the Mann-Whitney test. *P*-value levels are presented as **P* < 0.05 and ***P* < 0.01. M/S: Moderate/Severe.

Supplementary figure 8. Analysis of functional responsiveness of γδ T cells after stimulation for 6 hours with PMA/Ionomycin. (a, b) The Frequency of activation marker (CD69 and NKG2D) and cytokine production (IFN-γ, TNF, and MIP-1β) by γδ T cells, Vδ1, and Vδ2 subsets (n = 15). (c) The Proportion of cytokine production by NKG2D^+^ γδ T cells and NKG2D^-^ γδ T cells (n= 13). Each bar chart is shown as median. The significant differences were compared using the Mann-Whitney test. *P*-value levels are presented as **P* < 0.05, ***P* < 0.01 and ****P* < 0.001.
